# P300 as a Potential Indicator in the Evaluation of Neurocognitive Disorders After Traumatic Brain Injury

**DOI:** 10.3389/fneur.2021.690792

**Published:** 2021-09-09

**Authors:** Haozhe Li, Ningning Li, Yan Xing, Shengyu Zhang, Chao Liu, Weixiong Cai, Wu Hong, Qinting Zhang

**Affiliations:** ^1^Shanghai Key Laboratory of Forensic Medicine, Key Lab of Forensic Science, Ministry of Justice, Shanghai Forensic Service Platform, Academy of Forensic Science, Shanghai, China; ^2^Hongkou District Mental Health Center, Shanghai, China; ^3^Shanghai Mental Health Center, Shanghai Jiao Tong University School of Medicine, Shanghai, China

**Keywords:** neurocognitive disorders, traumatic brain injury, event-related potential, P300, daily life ability, social function

## Abstract

Few objective indices can be used when evaluating neurocognitive disorders after a traumatic brain injury (TBI). P300 has been widely studied in mental disorders, cognitive dysfunction, and brain injury. Daily life ability and social function are key indices in the assessment of neurocognitive disorders after a TBI. The present study focused on the correlation between P300 and impairment of daily living activity and social function. We enrolled 234 patients with neurocognitive disorders after a TBI according to ICD-10 and 277 age- and gender-matched healthy volunteers. The daily living activity and social function were assessed by the social disability screening schedule (SDSS) scale, activity of daily living (ADL) scale, and scale of personality change following a TBI. P300 was evoked by a visual oddball paradigm. The results showed that the scores of the ADL scale, SDSS scale, and scale of personality change in the patient group were significantly higher than those in the control group. The amplitudes of Fz, Cz, and Pz in the patient group were significantly lower than those in the control group and were negatively correlated with the scores of the ADL and SDSS scales. In conclusion, a lower P300 amplitude means a greater impairment of daily life ability and social function, which suggested more severity of neurocognitive disorders after a TBI. P300 could be a potential indicator in evaluating the severity of neurocognitive disorders after a TBI.

## Introduction

In recent years, traumatic brain injury (TBI) has been noticeably increasing, which leads to millions of people suffering from long-term disabilities (1). Some patients with a craniocerebral injury tend to have an abnormal behavior and cognitive and social function impairments, and about 21.7% patients will exhibit symptoms of mental disorders (2). According to the severity of TBI, a psychiatric diagnosis was found in about 49% of patients with severe and moderate TBI and 34% of patients with mild TBI, compared to 18% in the control group (3). Therefore, more attention should be paid on neurocognitive disorders after a TBI, which result in large social and economic burden (4). However, many patients with TBI may not receive an adequate follow-up therapy because of their lack of a consistent concern of the symptoms (5). Some progress has been made in the relationship between TBI and cognitive impairment, but the pathogenesis of neurocognitive disorders after a TBI was still uncertain (6, 7). Few effective methods can be used in the assessment of neurocognitive disorders after a TBI. Although computed tomography (CT) scanning and magnetic resonance imaging (MRI) can recognize a structural injury in the brain, the results of CT scanning and MRI have little effect on evaluating the severity of functional injury, such as neurocognitive disorders after a TBI. No obvious brain structural changes at the time of assessment could be found in ~67% patients with mild traumatic brain injury, but some patients do exhibit an abnormal behavior and a cognitive impairment (8). Although more details of brain structure and function could be detected by using functional neuroimaging in patients with traumatic brain injury, the results of functional neuroimaging cannot be well-applied in the assessment of neurocognitive disorders after a TBI (9, 10).

Event-related potentials (ERPs) are neurophysiological markers that can reflect human information processing and provide an index of cognitive function, such as memory, attention, concentration, and problem-solving (11–13). The P300 wave is an ERP component recorded by an electroencephalogram (EEG). The P300 is a positive potential that peaks ~250–500 ms after stimulus onset and often serves as an index of cognitive function in the process of decision-making (14–16). Changes in P300 latency and amplitude have been found in patients with mild cognitive impairment (17–20). A longer latency and a smaller amplitude of P300 were demonstrated in schizophrenic patients compared with healthy people. In particular, a longer latency was revealed in patients with the paranoid subtype of schizophrenia than with other subtypes of schizophrenia (21–23). It has been reported that P300 could reflect the improvement of cognition when using atypical antipsychotics in schizophrenic patients (24–27). Prolonged P300 latency was reported in patients with major depressive disorders (MDD) (28); and furthermore, P300 latency was found to be significantly different between mild, moderate, and severe MDD (29). The P300 latency tended to be longer, and the amplitude tended to be smaller in patients with cerebral contusion and intracerebral hematoma (30, 31). Negative changes in ERPs have been found in patients with mild brain injury (32). In recent studies, P300-based brain–computer interfaces provide an additional communication channel for individuals with communication disabilities and new control options for patients with impairments of eye movement or vision (33–35).

Daily life ability and social function are key indices in the assessment of a TBI (36–38). The scales of social disability screening schedule (SDSS) and activity of daily living (ADL) were proved to be associated with the severity of neurocognitive disorders after a TBI (39). Some criteria for the assessment of psychiatric impairment in patients with neurocognitive disorders after a TBI also list daily living activity and social function as important evaluation standards, such as the “Guideline for Assessment of Impairment in the Injured” in China (40), the AMA Guides to the Evaluation of Permanent Impairment, 6th Revision, by the American Medical Association (41), and the AAPL Practice Guideline for the Forensic Evaluation of Psychiatric Disability by the American Association of Psychiatry and Law (42).

In the current study, we aimed to investigate the correlation between visual P300 and impairment of daily living activity and social function, which, to some extent, could reflect the severity of neurocognitive disorders after a TBI.

## Materials and Methods

### Participants

**Figure 1:**
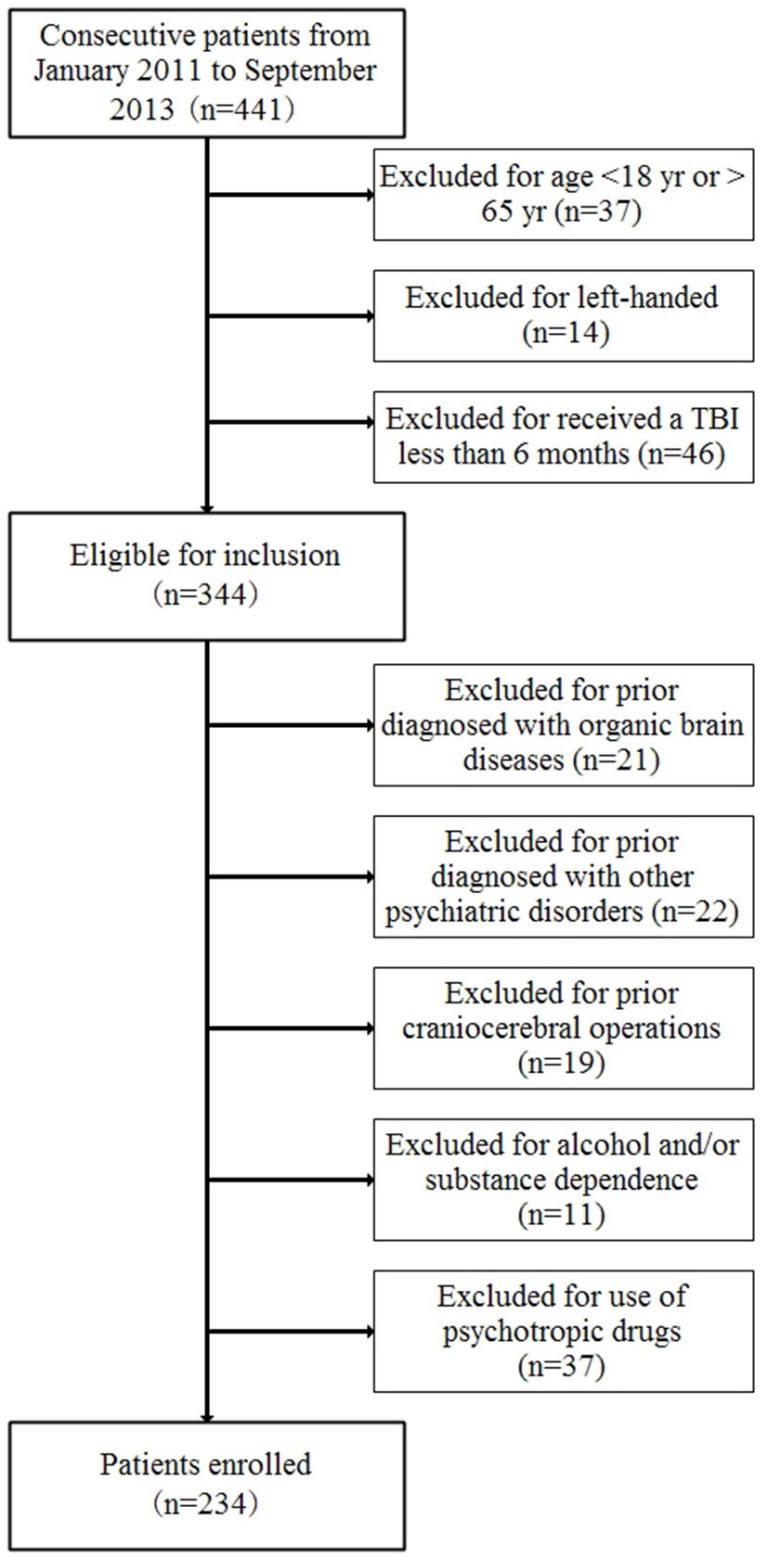
Cohort diagram of patients enrolled.

According to the International Statistical Classification of Diseases and Related Health Problems, Tenth Revision (ICD-10) criterion F07 and Z87.820, 234 patients with neurocognitive disorders after a TBI were enrolled from January 2011 to September 2013 in the Academy of Forensic Science (Figure 1). The inclusion criteria were as follows: (1) age from 18 to 65 years old, (2) a diagnosis of neurocognitive disorders after a TBI according to the ICD-10 criteria, (3) right-handed, and (4) received a traumatic brain injury at least 6 months previously. The exclusion criteria included whether the patient had ever been diagnosed with other psychiatric disorders and organic brain diseases or experienced craniocerebral operations before the current TBI or with alcohol and/or substance dependence or use of psychotropic drugs. The control group comprised of 277 age- and gender-matched healthy volunteers who were made up by staff and students in the Academy of Forensic Science. All the healthy controls were volunteers and met the following inclusion and exclusion criteria: (1) age from 18 to 65 years old, (2) right-handed, (3) no mental disorders, (4) no family history of mental disorders, (5) no organic brain diseases, (6) no significant medical history, and (7) no use of psychotropic drugs. In addition, the subjects were also excluded if there had been any lack of consensus.

The study was approved by the ethics committee of the Academy of Forensic Science. All the methods and procedures of the study were performed in accordance with the criteria laid out by the Declaration of Helsinki and other relevant national and international requirements for human research. All participants signed a written informed consent.

### Scales and Intelligence Quotient Assessment

Self-reported clinical research forms were used to collect the demographic characteristics and experimental data. The ADL scale, SDSS scale, and scale of personality change following a traumatic brain injury were filled out according to the medical records and interviews by the researchers.

The daily life ability and social function were mainly evaluated by ADL scale, SDSS scale, and scale of personality change following a traumatic brain injury. The ADL scale lists 14 items, and each item is scored from 1 to 4, which provides a simple and feasible method to evaluate the severity of impairment of daily living ability. If the total scores are more than 16, the patients are deemed to have impairment of daily life ability. The higher the score means more severe impairment of daily living ability. The SDSS scales was originally developed by the Disability Assessment Schedule of World Health Organization. The SDSS scale is composed of 10 items, which is used to assess social function. Each item is scored from 0 = healthy or very minor defects to 2 = severe defect. If the total scores are more than 2, it means that the patients have a social dysfunction. A higher score means more severe impairment of a social function. Cronbach's α was 0.88–0.92 (43). The scale of personality change following a traumatic brain injury includes 19 items, which can be used to evaluate the level of personality change (44, 45). Each item is scored from 0 to 3: zero point means absence, one point means occasionally, two points mean sometimes, and three points mean frequently. If the total scores are 7–14, it means a mild personality change, 15–21 means a moderate personality change, and 22–57 means a severe personality change. The Kappa value of diagnostic consistency was 0.86. The sensitivity of scale was 95.5%, and the false positive rate was 2.4% (44).

The Wechsler Intelligence Test (Chinese version) was used to collect the intelligence quotient (IQ) levels of the subjects.

### Stimuli and Procedures of Visual P300

The P300 waves were recorded by a Brain Master neurofeedback device (BrainMaster Technologies, Inc.). During the study, the subjects were seated in a dark and quiet room with an ambient temperature between 22 and 24°C. The subjects were instructed to be relaxed, keep awake, and focus on the screen. ERPs were recorded according to the 10–20 system. The reference electrodes were linked to the mastoid process. The impedance of Ag/AgCl surface electrodes was maintained below 5 kΩ. The oddball paradigm was used with the target stimuli randomly interspersed among the non-target stimuli. The target stimuli (circle, 5 cm in diameter) occurred 20% of the time and the non-target stimuli (square, 5 cm in length) 80%, with 300 stimuli presented in total. Each stimulus lasted 500 ms, with a white shape on a black background, and the interval of each stimulus was 500 ms. The subjects were required to press the F button on the keyboard when target stimuli (circle) occurred and press the J button when non-target stimuli (square) occurred.

**Figure 2:**
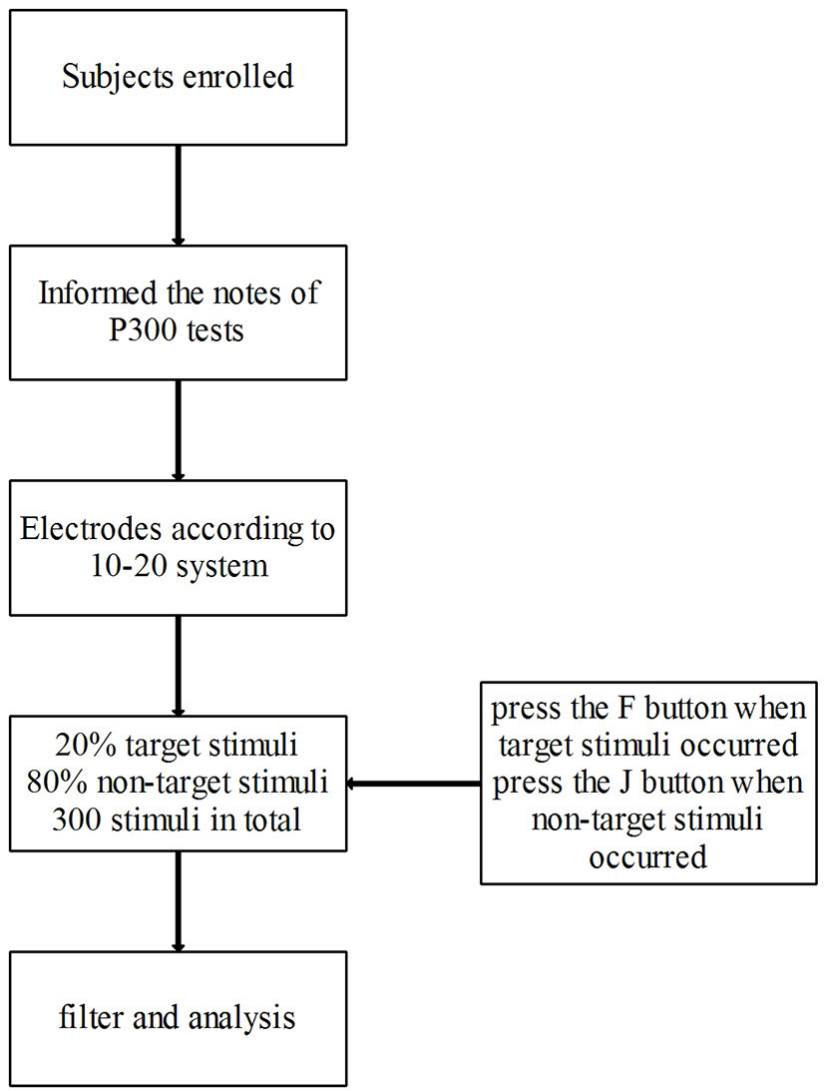
The paradigm of stimuli and procedures of visual P300.

The P300 component was defined as the positive peak with a latency of 300–500 ms at each electrode after the time of target stimuli onset. The amplitudes were measured as the maximum value of peaks to a pre-stimulus baseline, and the latency was measured from the start of the stimulus. The stimulus artifact of electro-oculogram and electromyography, high-amplitude noise (≥75 μV), and bad block were removed before the analysis. The EEG data were analyzed by the device mentioned above (Figure 2).

### Statistical Analyses

Data were analyzed by IBM Statistical Product and Service Solutions, version 22.0 (IBM SPSS 22.0). The statistically significant level was set as *P* < 0.05. All data were presented as mean ± standard deviation (SD). Categorical data were compared by chi-square tests. Student's *t*-test was used to compare the variables between patients and healthy controls. Pearson's correlation analysis was used to calculate the correlation between parameters.

## Results

From the 234 patients with neurocognitive disorders after a TBI, 227 (97.01%) completed the study, and seven patients could not cooperate well and did not complete the study. All 277 healthy volunteers accomplished the study. As revealed in Table 1, no statistically significant difference was found between patients and controls in age and gender. The scores of ADL scale, SDSS scale, and scale of personality change following a TBI in the patient group were significantly higher than those in the control group (*P* < 0.001). The IQ in the patient group was significantly lower than that in the control group (*P* < 0.001). The button press accuracy of the target stimuli and the non-target stimuli in the patient group was significantly lower than that in the control group (*P* < 0.05). The response time of button press of the target stimuli and the non-target stimuli in the patient group was significantly longer than that in the control group (*P* < 0.05).

**Table 1:** Demographic and clinical characteristics between patients with neurocognitive disorders after TBI and controls.

**Parameter**	**TBI (*n* = 227)**	**Controls (*n* = 277)**	***p*** **-value**
Gender			0.107^a^
Male	166	183	
Female	61	94	
Age in years	44.43 ± 13.88	46.03 ± 15.78	0.131
Course of disease in months	12.35 ± 6.44		
IQ	66.97 ± 13.00	85.20 ± 12.48	<0.001^b^
ADL scale scores	19.25 ± 2.78	15.36 ± 1.61	<0.001^b^
SDSS scale scores	9.69 ± 2.94	1.73 ± 2.26	<0.001^b^
Scale of personality change following TBI scores	11.97 ± 4.66	7.22 ± 3.44	<0.001^b^

*Except gender, other data were presented as mean ± SD*.

*IQ, intelligence quotient; ADL, activity of daily living; SDSS, social disability screening schedule; TBI, traumatic brain injury*.

a *Chi-square test; other statistics: Student's t-test*.

b *Statistically significant differences*.

**Figure 3:**
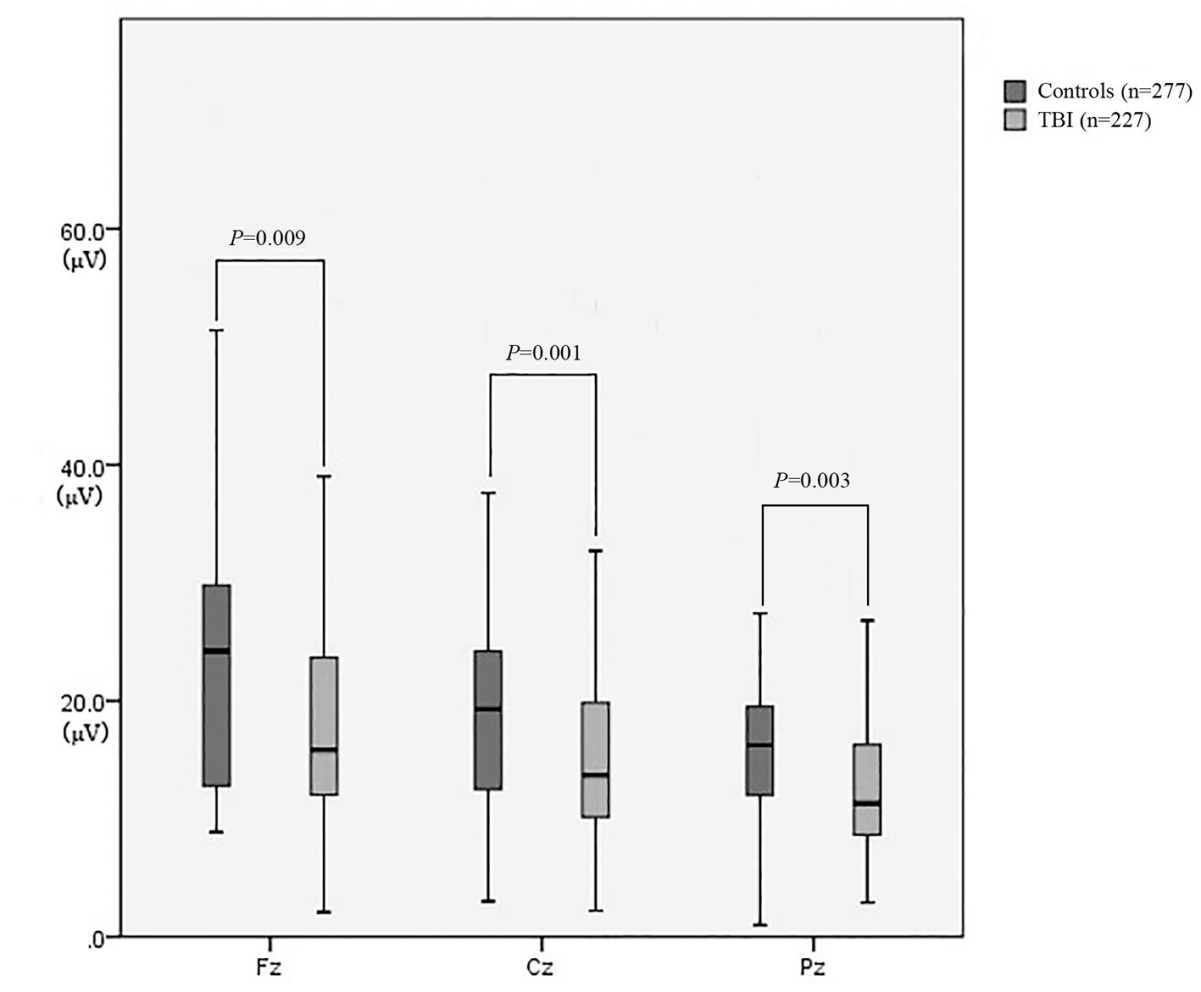
The amplitude of P300 between patients with neurocognitive disorders after TBI and controls.

**Figure 4:**
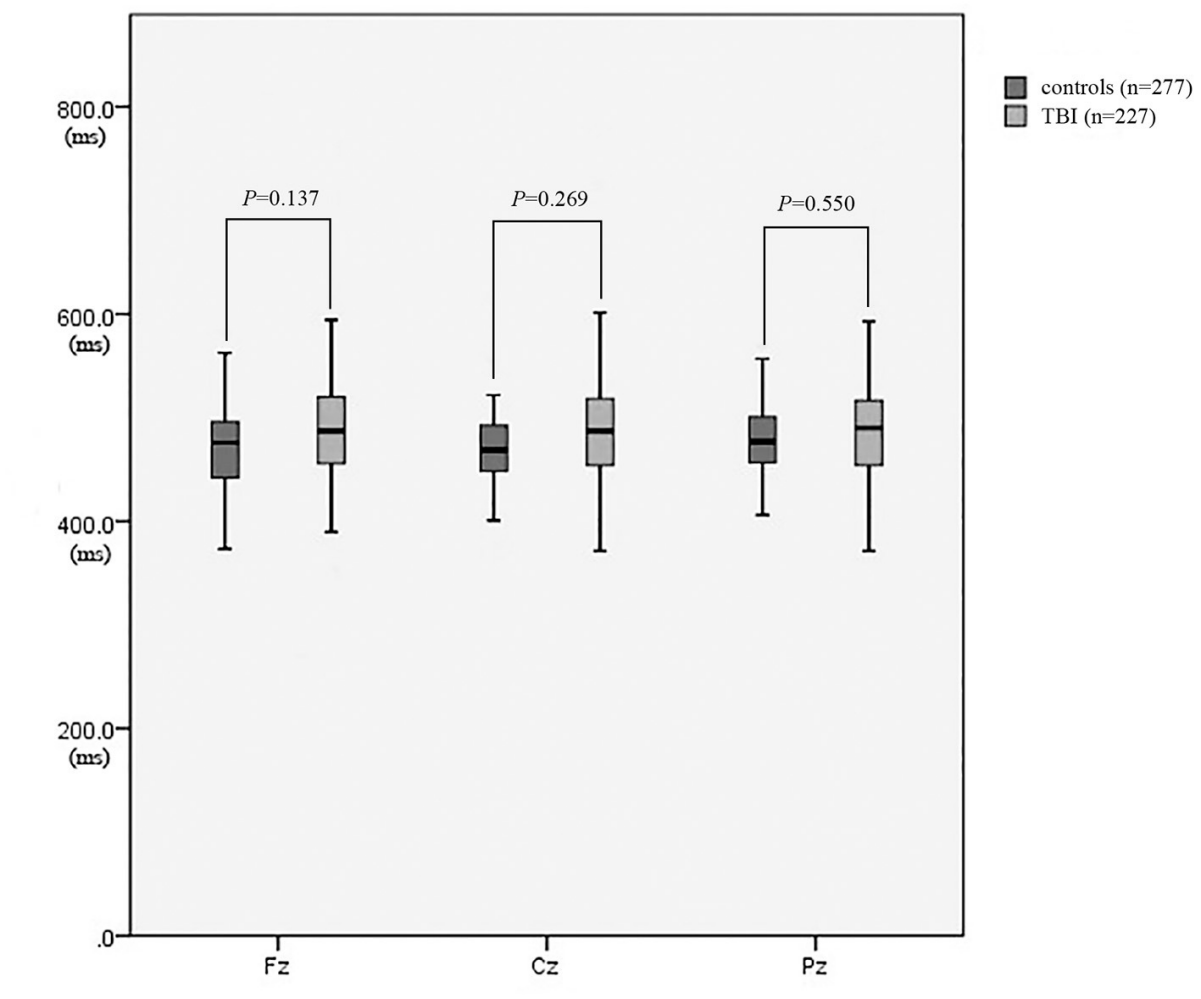
The latency of P300 between patients with neurocognitive disorders after TBI and controls.

As revealed in Table 2, the amplitudes of Fz, Cz, and Pz in the patient group were significantly lower than those in the control group (*P* = 0.009, *P* = 0.001, and *P* = 0.003, respectively) (Figure 3). There was no significant difference in the latencies of Fz, Cz, and Pz between the patient group and the control group (*P* > 0.05; Figure 4).

**Table 2:** The results of P300 between patients with neurocognitive disorders after TBI and controls.

**Parameter**	**TBI (*n* = 227)**	**Controls (*n* = 277)**	***p*** **-value**
**Amplitude (μV)**			
Fz	18.96 ± 10.52	24.90 ± 13.85	0.009^a^
Cz	15.15 ± 6.80	20.21 ± 11.03	0.001^a^
Pz	12.73 ± 5.55	16.22 ± 6.40	0.003^a^
**Latency (ms)**			
Fz	490.04 ± 57.76	472.88 ± 47.35	0.137
Cz	488.10 ± 57.18	475.63 ± 49.52	0.269
Pz	488.44 ± 54.53	481.94 ± 43.31	0.550
**Accuracy of button press (%)**			
Target stimuli	54.45 ± 26.09	69.12 ± 22.38	0.003^a^
Non-target stimuli	72.31 ± 24.48	82.60 ± 18.32	0.022^a^
**Response time of button press (ms)**			
Target stimuli	724.90 ± 180.95	604.48 ± 161.74	<0.001^a^
Non-target stimuli	644.49 ± 159.86	571.82 ± 135.91	0.014^a^

*TBI, traumatic brain injury*.

*Data were presented as mean ± SD. Data were calculated by Student's t-test*.

a *Statistically significant differences*.

As revealed in Table 3, the amplitudes of Fz, Cz, and Pz in the patient group were negatively correlated with the scores of the ADL and SDSS scales (*P* < 0.05). There was no correlation between the amplitudes of Fz, Cz, and Pz and the scores of the scale of personality change following a TBI (*P* > 0.05). The latencies of Fz, Cz, and Pz showed no significant correlation between the scores of the ADL scale, SDSS scale, and scale of personality change following a TBI (*P* > 0.05).

**Table 3:** Correlations between P300 and scale scores in patients with neurocognitive disorders after TBI.

**Parameter**	**ADL scale**		**SDSS scale**		**Scale of personality change following TBI**	
	***p*** **-value**	***r*** **-value**	***p*** **-value**	***r*** **-value**	***p*** **-value**	***r*** **-value**
**Amplitude (μV)**						
Fz	0.010^a^	−0.182	0.035^a^	−0.154	0.418	−0.057
Cz	0.002^a^	−0.213	0.003^a^	−0.214	0.248	−0.081
Pz	0.001^a^	−0.244	0.003^a^	−0.219	0.276	−0.077
**Latency (ms)**						
Fz	0.048	0.140	0.147	0.107	0.056	0.135
Cz	0.157	0.099	0.328	0.071	0.066	0.128
Pz	0.206	0.090	0.482	0.052	0.181	0.095

*ADL, activity of daily living; SDSS, social disability screening schedule; TBI, traumatic brain injury*.

*Data were calculated by Pearson's correlation analysis*.

a *Statistically significant differences*.

## Discussion

The severity of neurocognitive disorders after a TBI was associated with lots of factors and difficult to be evaluated. The current study focused on how to evaluate the severity of mental disorders due to TBI through P300. The result showed that the intelligence quotients of patients were lower than those of healthy controls, which meant that patients with neurocognitive disorders after a TBI might suffer a mild intelligence impairment. The accuracy and response time of button press between the patient and control groups were also objective indicators which could reflect the levels of cognitive impairment in patients with neurocognitive disorders after a TBI. Mild intelligence impairment was one of the main symptoms of neurocognitive disorders after a TBI. P300 has been extensively studied in mild cognitive impairment and was regarded as an ideal marker for assessing brain function in cognitive diseases (46, 47). The assessment of P300 amplitudes and latencies might offer valuable information in patients with cognitive impairment (48)—for example, attenuated amplitude and longer latency (19, 49)—which was partly consistent with our findings that the amplitudes of patients were lower than those in healthy controls. The amplitudes of P300 reflect the allocation of attentional resources and the ability of information processing; therefore, the decrease of amplitude may mean a decreased ability of information processing, decreased neuronal efficiency, and impairment of attention (50–53). According to our findings, patients with neurocognitive disorders after a TBI may suffer from impairment of attention and information processing ability, which may lead to the impairment of daily life ability and social function. The latencies of P300 in the current study showed no significant difference between patients and healthy controls. The latencies of P300 were regarded to be related to cognitive performance (51). The latencies of P300 were found to have a significant difference between patients with mild TBI and patients with moderate and severe TBI (54). Although the current study found that the response time of button press in the patient group was significantly longer than those in the control group, a previous study showed that latency in behavioral response time was not correlated with the latency of P300 (55). Therefore, the reason why the current study found no significant difference between patients and healthy controls might be that the cognitive impairment of patients enrolled was mild and the latencies of P300 were associated with the severity of cognitive impairment.

The current study revealed the impairment of daily life ability and social function in patients with neurocognitive disorders after a TBI and the correlation between P300 amplitudes and daily life ability and social function. The impairment of daily life ability and social function was also found in patients with a TBI (56, 57). The daily life ability and social function were regarded as being more appropriate than symptoms when assessing a TBI and could comprehensively assess a TBI (56–58). A further study identified the value of evaluating the daily life ability and social function in patients with neurocognitive disorders after a TBI (39). According to our findings, the lower amplitudes of P300 mean a greater impairment of daily life ability and social function, which suggested more severity of neurocognitive disorders after a TBI. The main reason might be that patients with neurocognitive disorders after a TBI suffered the impairment of ability of information processing, which was mentioned above.

Personality change is also one of the main symptoms in neurocognitive disorders after a TBI, which would lead to the impairment of social function. The current study found that patients with neurocognitive disorders after a TBI did exhibit a personality change, but P300 showed no correlation with the severity of personality change. Personality change in neurocognitive disorders after a TBI includes apathy, affective lability, and aggression (39). Personality change was an acquired disease after a TBI, stress, etc., and the reason of personality change after a TBI is still unknown (59). Failure to regulate emotions might result in a personality change (60). Dysfunction of the frontal lobe was also regarded to be associated with a personality change (61). The P300 amplitude was reported as an abnormally enhanced amplitude in borderline personality disorder (62) and a decrement at the anterior electrode sites in antisocial personality disorder (63), which meant that P300 could also, to some extent, reflect a personality disorder. Therefore, P300 might be one of the indicators which could reveal a personality change in patients with neurocognitive disorders after a TBI but could not show the severity of a personality change.

## Limitation

Some limitations should be clarified. First, considering the cooperation of subjects during the ERP tests, the symptoms of patients enrolled in the study were relatively mild. Second, the patients enrolled in the study suffered a mild TBI, and the aim of the study was to explore the severity of neurocognitive disorders after a TBI, so the injury areas were not analyzed. Third, due to the limitation of the ERP device and no analysis of injury areas, only three individual electrodes were used, and the differentiation of P3a and P3b could not be distinguished.

## Conclusion

Impairment of daily life ability and social function and personality change were found in neurocognitive disorders after a TBI. The P300 amplitude was positively correlated with an impairment of daily life ability and social function. A lower P300 amplitude means a greater impairment of daily life ability and social function, which suggested more severity of neurocognitive disorders after a TBI. Therefore, P300 could be a potential indicator in evaluating the severity of neurocognitive disorders after a TBI.

## Data Availability Statement

The datasets generated for this study are available on request to the corresponding author.

## Ethics Statement

The studies involving human participants were reviewed and approved by the ethics committee of the Academy of Forensic Science. The patients/participants provided their written informed consent to participate in this study.

## Author Contributions

HL and NL participated in the design of the study and drafted the manuscript. YX performed the experiments. SZ and CL enrolled patients and healthy volunteers and performed statistical analyses. WC participated in the design of the study and helped in proofreading. WH and QZ conceived the study protocol and participated in the design and coordination of the study. All authors contributed to the final text and approved it.

## Funding

This study was supported by the National Natural Science Foundation of China (81801881 and 81001354), the National Key R&D Program of China (2016YFC0800701), and the Science and Technology Committee of Shanghai Municipality (20DZ1200300, 19DZ2292700, and 17DZ2273200).

## Conflict of Interest

The authors declare that the research was conducted in the absence of any commercial or financial relationships that could be construed as a potential conflict of interest.

## Publisher's Note

All claims expressed in this article are solely those of the authors and do not necessarily represent those of their affiliated organizations, or those of the publisher, the editors and the reviewers. Any product that may be evaluated in this article, or claim that may be made by its manufacturer, is not guaranteed or endorsed by the publisher.
